# Safer Type 1 Diabetes Care at Home: SEIPS-based Process Mapping with Parents and Clinicians

**DOI:** 10.1097/pq9.0000000000000649

**Published:** 2023-05-22

**Authors:** Eric S. Kirkendall, Patrick W. Brady, Sarah D. Corathers, Richard M. Ruddy, Catherine Fox, Hailee Nelson, Tosha B. Wetterneck, Isabelle Rodgers, Kathleen E. Walsh

**Affiliations:** From the * Center for Healthcare Innovation, Wake Forest University School of Medicine, Winston-Salem, N.C.; † Center for Biomedical Informatics, Wake Forest University School of Medicine, Winston-Salem, N.C.; ‡ Department of Pediatrics, Wake Forest University School of Medicine, Winston-Salem, N.C.; § Division of Hospital Medicine, Cincinnati Children’s Hospital Medical Center, Cincinnati, Ohio; ¶ James M. Anderson Center of Health Systems Excellence, Cincinnati Children’s Hospital Medical Center, Cincinnati, Ohio; ‖ Department of Pediatrics, University of Cincinnati College of Medicine, Cincinnati, Ohio; ** Division of Endocrinology, Cincinnati Children’s Hospital Medical Center, Cincinnati, Ohio; †† Division of Emergency Medicine, Cincinnati Children’s Hospital Medical Center, Cincinnati, Ohio; ‡‡ Department of Medicine, Division of General Internal Medicine, University of Wisconsin School of Medicine and Public Health, Madison, Wis.; §§ Division of General Pediatrics, Harvard Medical School, Boston, Mass.; ¶¶ Department of Pediatrics, Boston Children’s Hospital, Boston, Mass.

## Abstract

**Introduction:**

The limited data indicate that pediatric medical errors in the outpatient setting, including at home, are common. This study is the first step of our *Ambulatory Pediatric Patient Safety Learning Lab* to address medication errors and treatment delays among children with T1D in the outpatient setting. We aimed to identify failures and potential solutions associated with medication errors and treatment delays among outpatient children with T1D.

**Methods:**

A transdisciplinary team of parents, safety researchers, and clinicians used Systems Engineering Initiative for Patient Safety (SEIPS) based process mapping of data we collected through in-home medication review, observation of administration, chart reviews, parent surveys, and failure modes and effects analysis (FMEA).

**Results:**

Eight (57%) of the 14 children who had home visits experienced 18 errors (31 per 100 medications). Four errors in two children resulted in harm, and 13 had the potential for harm. Two injuries occurred when parents failed to treat severe hypoglycemia and lethargy, and two were due to repeated failures to administer insulin at home properly. In SEIPS-based process maps, high-risk errors occurred during communication between the clinic and home or in management at home. Two FMEAs identified interventions to better communicate with families and support home care, especially during evolving illness.

**Conclusion:**

Using SEIPS-based process maps informed by multimodal methods to identify medication errors and treatment delays, we found errors were common. Better support for managing acute illness at home and improved communication between the clinic and home are potentially high-yield interventions.

## INTRODUCTION

After almost two decades of dedicated attention, we are beginning to realize some improvements in healthcare safety for hospitalized children.^1^ Yet, most healthcare is provided in the clinic and at home. In these contexts, limited epidemiologic data exist, and researchers have conducted few intervention studies. Two in 5 children with chronic disease suffer from a medication error in the outpatient setting, including the home.^2,3^ Of these, 3.6% are injured due to these errors: the same injury rate as hospitalized children.^3^ Children in outpatient settings, including at home, are especially vulnerable to medical errors for many reasons, including fragmented outpatient care, the child’s limited ability to communicate evolving symptoms, and failed coordination between caregivers. For example, poison control centers receive 1 phone call every 8 minutes about pediatric out-of-hospital overdose.^4^ In another example, telephone triage nurses miss symptoms of serious illness 64% of the time.^5^ The field of ambulatory pediatric patient safety has substantial gaps to close.

This study focused on outpatient children with Type 1 Diabetes (T1D), in which there is a substantial risk of medication errors that can result in dangerous under- or overdosing of insulin. Among the 165,000 children with type 1 diabetes (T1D) nationally, only 17% achieve the American Diabetes Association goal for HbA1c.^6,7^ Among individuals with T1D, the leading cause of death before age 30 is acute complications (eg, severe hypoglycemia, diabetic ketoacidosis), and 5% of deaths are attributable to a sudden unexplained death syndrome in young people with T1D.^8^ These observations highlight the complicated and nuanced challenges of balancing clinical goals and safety through increasing awareness and knowledge, optimizing medication regimens, and promoting adherence.

This study is the first step of an ongoing project called *The Ambulatory Pediatric Patient Safety Learning Lab*, funded by AHRQ, which uses a user-centered design process to address medication errors and treatment delays among children with T1D in the outpatient and home setting. The user-centered design process includes problem analysis, design, development, implementation, and evaluation; this article describes the problem analysis aspect and is the first phase of a broader quality improvement effort. *We aimed to identify failures and potential mitigations to reduce medication errors and treatment delays among outpatient children with T1D.*

## METHODS

A transdisciplinary team of parents, safety researchers, and clinicians used Systems Engineering Initiative for Patient Safety (SEIPS) based process mapping, with data collected through in-home medication review, medication administration observation, parent surveys, and failure modes and effects analysis (FMEA). The SEIPS model was selected due to its safety-centeredness and scientific rigor (its validity, acceptance, and commonality in the patient safety community and literature). Our study team includes clinicians, parents, and researchers providing expertise in human factors, pediatric ambulatory patient safety, informatics, and patient care. A design firm provided consultation throughout the study. Our focus on ambulatory patient safety was well aligned with hospital priorities, and the priorities of health system leaders in quality and ambulatory care drove study aims. Collaborating with parents and clinicians, we focused on medication errors and treatment delays germane to ambulatory T1D.

### Setting and Subjects

The study occurred at a large pediatric academic health center with 1.5 million annual outpatient visits. The Diabetes Center has more than 2000 patients. The clinic is staffed by endocrinologists, nurses, certified diabetes care and education specialists, social workers, and psychologists. Epic (Verona, Wis.) was the electronic health record (EHR) system, and patients had access to MyChart, Epic’s patient portal.

A research coordinator approached parents (or guardians) of children 0–17 years with T1D for participation in the study during an endocrine clinic visit in 2019. In addition, the same coordinator recruited clinic staff for the FMEA in person or by email. All participants gave written informed consent.

### In-home Medication Reviews, Observations, Surveys, and Postvisit Chart Reviews

During home visits, a trained nurse reviewed medications, observed medication dosing and administration, interviewed the parent or guardian, and reviewed medical records focusing on outcomes 1 month following the visit. We collected demographics from the medical record or parent report and administered 2 surveys: the Problem Recognition in Illness Self-Management survey and the Newest Vital Sign survey.^9,10^

*Home visit procedures*. Before the visit, the nurse identified all current medications and doses from the medical record. Then, she reviewed all over-the-counter and prescription daily and as-needed medications in the home, focusing on insulin and tools used to determine insulin dosing. She also interviewed caregivers about each medication, including current dose and frequency, indication, problems with the medication (eg, dispensing errors in the past), and missed doses in the last week. She also assessed whether all the medications (including dose, route, frequency, formulation, and strength) from the EHR medication list were at home or whether any medications at home were not listed on the EHR list.

We developed a home visit observation tool based on the SEIPS 2.0 framework^11^ and informed by our prior research to record field notes on the home medication use process, including behaviors and tools used.^11,12^ The nurse asked questions about how the medication is given, such as why medicines may be given differently than prescribed and why medicines were not at home. To help identify errors, she compared the medication prescription to the bottle label, how families reported using it, and how they administered it. Using this method, inter-observer reliability for the detection of errors is excellent (K = 0.89).^12^

After the home visit, following established methods, the nurse reviewed all components of the patient’s ambulatory medical record for the subsequent month, including documented telephone interactions, medication orders administered in the clinic, home medication prescriptions, and all visit notes.^13^

*Error adjudication.* The nurse recorded detailed information about potential errors using a standardized form in REDCap.^14^ Medication doses that differed by 10% or more from the correct dose were considered errors.^15^ Two physicians made independent judgments about whether an error occurred and its severity (clinically trivial, potential for injury but did not injure, and injury; severity: significant, serious, life-threatening), including taking into account insulin-specific safety considerations.^3,16–18^ Inter-rater reliability for judgments about whether an error occurred was 100%. Whether the errors resulted in injury or had the potential for severe injury, the K was 0.62.

### Failure Mode and Effects Analysis (FMEA)

FMEA is a systematic team-based approach that seeks to understand how a process can fail and develop interventions to restructure the process.^19^ FMEAs, including parents and clinic staff, were used to provide a complex understanding of potential failures and a diverse set of solutions. The 2 FMEAs focused on 2 high-risk processes, selected by the study team of clinicians, researchers and parents to sample medication errors and treatment delays: (1) “A change in insulin dose due to abnormal glucoses at home” and (2) “Management of a sick child with T1D at home.” To teach the FMEA method to participants, the study team used real-world examples, simple language, and written materials at a third-grade reading level. Parents and clinic staff created draft process maps to ensure efficiency before each 2-hour FMEA meeting. Participants diagrammed the process, brainstormed failure modes, and proposed modes for each step in the process. To prioritize failure modes, we calculated risk priority numbers by multiplying participant Likert scale ratings of frequency of occurrence, the likelihood of detection, and clinician ratings of severity for each failure mode.^19,20^ The group proposed possible interventions for failure modes with the highest risk priority numbers.

### Analysis

Using descriptive statistics, we summarized demographics and error rates for the participants’ in-home visits (observation, medication review, surveys, and chart review). For the PRISM survey, items with mean scores > 2 were counted as self-management barriers.^9^ For the Newest Vital Sign survey, scores were calculated and reported as 0−1, 2−3, and 4−6.^21^ We used SEIPS-based process mapping to understand and analyze the diabetes home care processes. This method uses swim lane process mapping and, for each process step, outlines the system elements of people, tasks, tools and technologies, organization and environmental aspects and notes barriers and facilitators. Process maps were created using OmniGraffle Pro software (The Omni Group, Seattle, Wash.).

## RESULTS

From March to December 2019, we performed 14 home visits and 2 FMEAs with 8 parents and 4 clinic staff. The 14 children with T1D in the study were 4–17 years old and took 59 medications at home (Table 1). All parents used support tools at home, such as whiteboards or apps, to help manage their child’s T1D. Almost all children (n = 12) had multiple people responsible for medication management, with an average of 2 people (min 1, max 3) working together to manage their T1D. Five children had people who sometimes administered their medications but did not regularly attend clinic visits. Half of the parents (n = 7) reported that they had previously given the same medicine or missed a dose in the home due to errors in communication between in-home caregivers (eg, 2 parents).

**Table 1.** Demographics of Home Visits

	Total Patients (N = 14)
Medications, n, median, (min, max)	59, 4, (1–7)
Women, n (%)	5 (36%)
Insurance type, n (%)	5 (36%)
Race, n (%)	
White/Caucasian	9 (64%)
Black or African American	5 (36%)
Children with multiple Individuals administering meds n (%)	12 (86%)
Primary person responsible administering child’s meds n (%)	
Mom	8 (57%)
Dad	0 (0%)
Child (self-administration)	6 (43%)
Other individuals aside from primary caregiver administering meds n (%)	
Primary caregiver + mom	2 (14%)
Primary caregiver + dad	2 (14%)
Primary caregiver + child	3 (21%)
Primary caregiver + siblings	2 (14%)
Primary caregiver + mom and dad	1 (7%)
Primary caregiver + school nurse	1 (7%)
Primary caregiver + dad and siblings	1 (7%)
Only primary caregiver administers medications	2 (7%)
Parents who need help reading materials from physician or pharmacy n (%)	
Never	9 (64%)
Rarely	4 (29%)
Sometimes	1 (7%)
Often	0 (0%)
Always	0 (0%)
Parents newest vital sign health literacy score+ n (%)	
1−2 (greater than 50% of limited literacy)	1 (7%)
2−3 (possibility of limited literacy)	0 (0%)
4−6 (adequate literacy)	13 (93%)
Use support tools (ie, calendar) to manage child’s medications n (%)	14 (100%)
PRISM Barrier*+	
Factor 1. understanding/organizing care, n (%)	
Adolescent (n = 8)	5 (63%)
Parent of an adolescent (n = 7)	3 (43%)
Parent of a child (n = 6)	3 (50%)
Factor 2. regimen pain and bother	
Adolescent	4 (50%)
Parent of an adolescent	7 (100%)
Parent of a child	4 (67%)
Factor 3. denial	
Adolescent	0 (0%)
Parent of an adolescent	6 (86%)
Parent of a child	5 (83%)
Factor 4. healthcare term interactions	
Adolescent	1 (13%)
Parent of an adolescent	1 (14%)
Parent of a child	4 (67%)
Factor 5. family interactions	
Adolescent	5 (63%)
Parent of an adolescent	4 (57%)
Parent of a child	0 (0%)
Factor 6. peer interactions	
Adolescent	4 (50%)
Parent of an adolescent	6 (86%)
Parent of a child	0 (0%)

*Problem Recognition in Illness Self-management (PRISM tool developed by Cox et al) to identify the barriers experienced by children with T1D and their parents. Adolescents take their own version of the PRISM, modified from the version their parents take. The tool consists of 25 items across 6 domains. A mean score of ≥ indicates the presence of barriers to self-management of diabetes in the corresponding domain with higher scores indicating that the corresponding factor has a greater impact on the regulation of blood glucose levels in patients with T1D.

+Surveys were completed by all participants. There was no missing data.

Eight (57%) of the 14 children who had home visits and chart reviews experienced 18 errors (31 per 100 medications) detected during the home visit or chart review (Table 2). Of these, 4 errors were harmful, and 13 had the potential to harm the child but did not. All 4 errors with harm were separate incidents that occurred for 2 patients with comorbid mental health issues. Two injuries required emergency department (ED) visits for severe hypoglycemia and lethargy, where the child did not receive glucagon or any oral carbohydrates at home for rescue therapy. Two others were repeated failures to administer insulin at home properly. Two errors with potential harm were considered life-threatening, and 6 were considered serious. These included patients with expired glucagon at home (families may choose not to use expired medications at all) and missed insulin doses or under-doses (eg, no carb correction, no glucose checks, did not change insulin pump, insulin overdose by a substitute school nurse, and failure to check ketones with high blood sugars). For example, a child had a glucose of 430 mg/dL at home, and the caregiver did not check ketones, even when reminded by the study nurse to do so. Of the 59 medications the 14 children took at home, 90% were accurately listed in the child’s chart, including 91% of prescriptions and 71% of over-the-counter medications. Among the 14 patients in the study, 9 (64%; 95% CI 34%–87%) had a completely accurate medication list.

**Table 2.** Medication Errors Identified among 14 Children with T1D using In-home Medication Review, Observation of Administration, Caregiver Interview, and Chart Review

Error Type	Example
**Treatment delay/omission** (N = 2)	Child received insulin but did not eat in the morning. Parent drove the child to the hospital when the glucose was low; did not use glucagon or food because did not know to do***Error with potential for harm; life threatening***
**Not checking ketones at home** (N = 1)	During the home visit, a child had a glucose over 400 but did not check ketones, even after being reminded by the nurse to do so***Error with potential for harm; serious***
**Not checking sugars at home** (N = 1)	School nurse called the clinic because glucose range from 50 to 380; no one at home is checking glucose at all***Error with potential for harm; serious***
**Insulin under dose or missed doses** (N = 7)	Adolescent misses putting his glucose or carb count in the pump for a bolus, and lets his pump run out of insulin, changing only with the next meal***Error with potential for harm; serious***
**Wrong technique** (N = 5)	Parent administered insulin pen but removed the pen immediately while pen was still dispensing the dose, causing some of the insulin to be on the skin***Error with potential for injury; significant***
**Expired glucagon** (N = 2)	Family only had expired glucagon at home***Error with potential for injury, life-threatening***

### Findings from FMEAs and Process Mapping

**Fig. 1.**
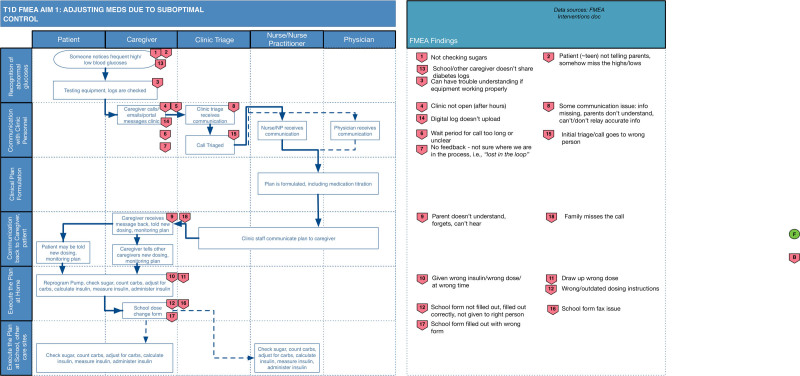
SEIPS-based process map with errors for adjusting medications due to suboptimal control.

For the high-risk process “A change in insulin dose due to abnormal glucoses at home,” the SEIPS-based process maps are shown in Figure 1. The highest risk failure modes were all failures in the first step of the process, “caregiver notices abnormal glucoses,” including not checking glucoses, not realizing glucose results were abnormal, adolescents telling parents glucoses were normal but actually not measuring it, and adolescents ignoring high glucoses, and/or did not tell adults about the elevation (Table 3). The FMEA attendees suggested interventions to make each glucose result, or the failure to check glucoses, visible to all stakeholders, alerting when values are high or low. These failure modes were higher risk (higher risk priority numbers) than errors in insulin administration because participants judged insulin administration errors to be less common.

**Table 3.** Highest Risk Failures Identified by 8 Parents and 4 Clinic Staff in 2 Separate Failure Modes and Effects Analyses (FMEAs)

**Process steps**	**Failure Mode**	**Intervention**
*FMEA #1. A change in insulin dose due to abnormal glucoses at home*		
Caregiver/patient notices frequent abnormal glucoses	No one is checking glucoses	Highly visible, prominent paper at home or app for recording glucoses
	Does not notice that the glucoses are abnormal	
	Adolescent patient forgets to tell anyone about the abnormal glucoses	
	When asked, adolescent does not tell the truth about the glucose levels to caregiver because she/he does not want to deal with it	
Caregiver contacts clinic	Correct information not relayed from parent to clinic	Tools (eg, handout, on hold message) that prep families for information needed, template for info needed, personal relationship with caregiver
	Clinic is not open, after hours, weekends and unable to get through to someone	Extend clinic hours, periodic after hour visits
*FMEA #2. Management of a sick child with T1D at home*		
Caregiver notes signs of illness	Overwhelming difficulty determining whether technology or illness as cause of symptoms and calling clinic while child is sick (eg, actively vomiting)	Simple just in time algorithm/process map that walks caregiver through troubleshooting, when to call (digital and paper-based options)
	Caregiver does not notice the illness until the child has gotten much worse later in the process, eg, a home-based diagnostic delay	A more active process for families and patients to engage in when patients first get sick, to help them understand how ill the patient is, if its T1D or something else (or both), etc.
	Unsure if sick (is it diabetes, or something else)	Parents suggested a peer consultant service
Clinic receives information from clinic about what to do	Information may be outdated (patient worsened while the clinic was processing and responding to original call from home)	A more active process for families and patients to engage in when patients first get sick, to help them understand how ill the patient is, if its T1D or something else (or both), etc.
Administer insulin	Cannot tell if ketones are trace/small/etc (hard to read)	Use blood ketone meters
Discussion between triage nurse and nurse practitioner	Caregiver unable to clearly explain what is going on or misses important information	A hold message or written handout/template of information needed when talking to clinic
Caregiver tells other caregivers about plan	The caregiver forgets to tell the other caregiver, or the other caregiver misunderstands and does not follow instructions (at home hand-off)	Visual, written information sent from clinic to home about management plan after phone call for caregiver to share with other caregivers

Failure modes were layered onto the SEIPS-based process maps.

FMEA, failure modes and effects analysis.

**Fig. 2.**
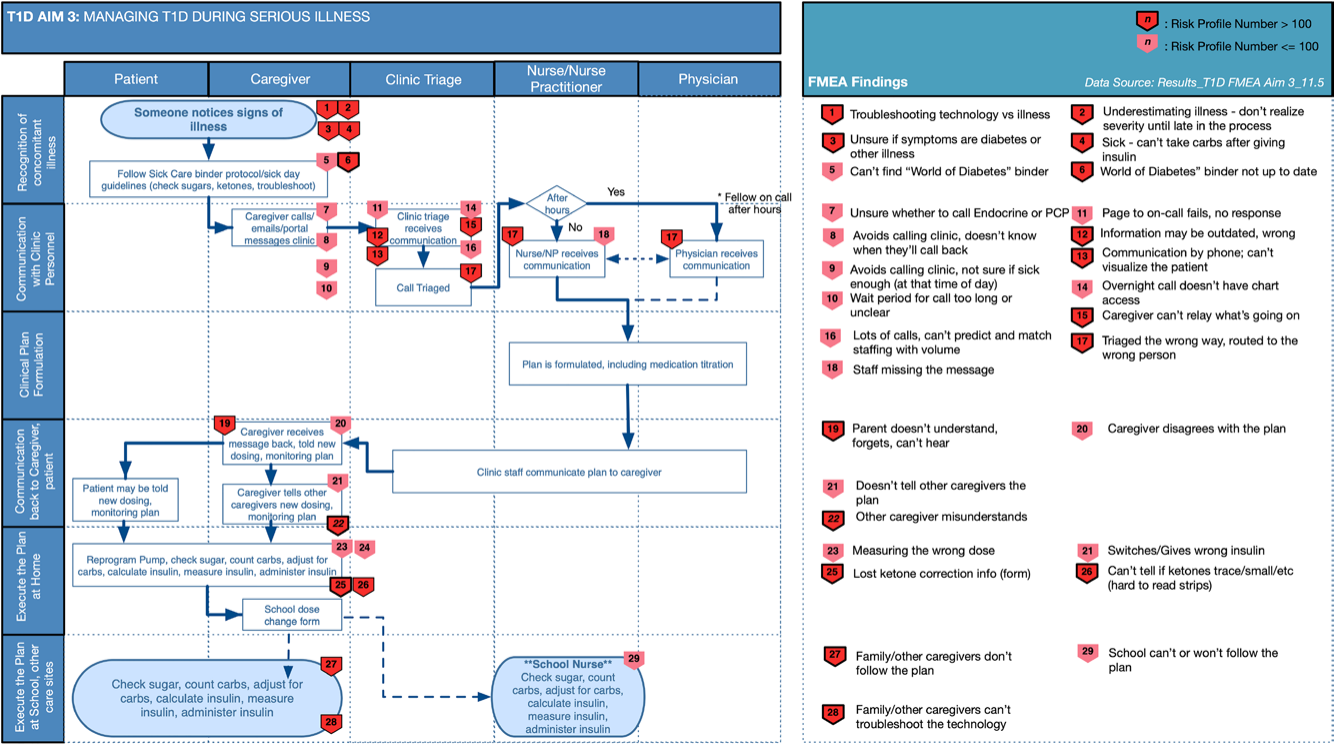
SEIPS-based process map with failure modes for managing type 1 diabetes during a concomitant serious illness*.

For the high-risk process, “Management of a sick child with T1D at home,” the final SEIPS-based process map is shown in Figure 2. The highest risk failure mode was “caregiver becomes overwhelmed troubleshooting the root cause of the symptoms – technology problem… vs. acute illness...” Participants noted that an acute illness and an insulin pump site failure could present similarly with high blood sugars. It was particularly challenging for parents to sort through both possibilities when dealing with the stress of a sick child. The group suggested a simple “just-in-time” algorithm, available digitally or on paper, for when the child is sick that walks the caregiver through troubleshooting technology, when to call the clinic, etc.

## DISCUSSION

Using home visits, chart review, and failure modes and effects analyses, we found several errors and failures associated with treatment delay and medication errors in the homes of children with T1D. SEIPS-based process mapping demonstrated most medication management and sick day process steps occur at home and facilitated identifying opportunities to improve coordination and support by health care workers for home care. Parents and clinicians identified interventions to share or shift work, including via additional, seamless communication with the clinic or additional support or training for caregivers at home.

This study focused on treatment delays and medication errors in the outpatient management of T1D, particularly at the interface between the health system and home. Most children (57%) who participated in chart review and home visits had errors, including 2 children with harm caused by errors. In previously published case studies of insulin administration errors, most errors were in administration at home.^22,23^ The rate of errors with potential for injury in our study was either similar to or higher than previous publications in other pediatric populations with chronic conditions.^2,3,13,24^ Children with T1D use a high-risk medication at home daily, with severe consequences for overdoses or under-doses.

The SEIPS-based process was particularly helpful in understanding the care processes of evolving illness at home, with support and advice from clinicians by phone or portal. Unfortunately, most children in our study had multiple caregivers helping administer their medications at home and in other locations, increasing the complexity and risk of error.^3^ This study highlights a need for improved scaffolding for caregivers administering medications to children with T1D, consideration for specific care processes at home to understand outpatient safety, and transitioning some of the responsibility of caregivers to other healthcare team members.

What interventions are needed to reduce errors in T1D management at home? This study suggests that increased frequency and quality of communication between healthcare professionals and caregivers is 1 way to shift some of the responsibility from caregivers to healthcare team members. Communication-based interventions have been successful in preventing errors in other settings.^25^ The 4 preventable harms identified in this study were in 2 children with serious mental illness, suggesting an important flag for all such patients, including proactive identification. Although these teens are already receiving intensive support, additional scaffolding, such as better-integrated social work staff in the T1D clinic or additional in-home support (eg, visiting nurse) for children with T1D and known mental illness, may provide critical support during this dangerous time. In addition, a specific intervention is needed to support refills of glucagon prescriptions (eg, a reminder from the pharmacy when the medication is out of date) and/or to ensure families always have glucagon available at home. Lastly, all interventions should be co-designed with patients and families, the healthcare team, and T1D clinical staff.

We have led previous studies on outpatient and home medication errors among children with cancer, sickle cell anemia, and epilepsy.^2,3,17,20,26^ Several common failures emerge from this research, employing chart review, home visits, and failure modes and effects analyses. Errors frequently occurred with dose changes, leading to failures to increase or decrease doses of chemotherapy, insulin, antiepileptic or hydroxyurea medications. These errors resulted from miscommunication between the clinician and the parent attending the clinic visit or miscommunication between in-home caregivers about changes in dose. To reduce home medication errors among children with cancer, Walsh et al co-produced with parents and clinicians a web-based Home Medication Support tool that parents and clinicians can use to update and track current chemotherapy doses.^27^ This study assessed usability and feasibility, and medication errors were detected during the chart review.

**Fig. 3.**
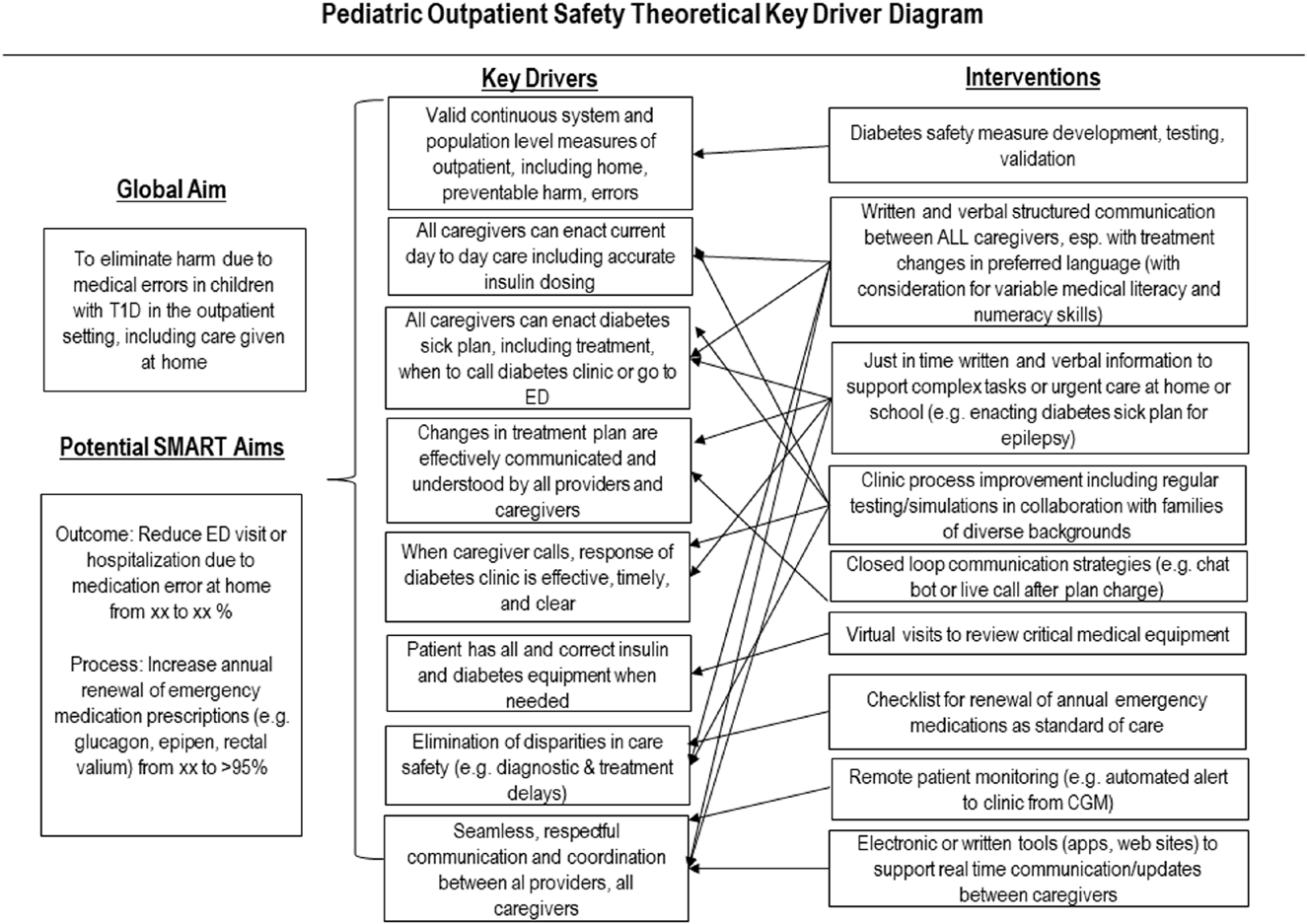
Key driver diagram, including aims and interventions.

Informed by findings in the current and these previous studies, we developed a key driver diagram (Fig. 3). Key drivers of improved patient safety in the outpatient setting, in which day-to-day care is provided at home, include seamless communication among and between all health system staff and caregivers. It also includes the ability of all caregivers to enact *both* routine day-to-day and sick-day care plans, including when and how to seek additional help from the clinic or in the emergency department. For ambulatory care to be safe, patients should have all needed medications and functional equipment.

The Institute for Healthcare Improvement workgroup on ambulatory safety, chaired by Drs. Bonner and Muething, highlight the need for measurement and reporting infrastructure to improve outpatient safety.^28^ Unfortunately, there is no established set of *measures* for outpatient pediatric adverse events that health systems can use to improve the safety of outpatient medication use. This deficit is a critical barrier to safe outpatient healthcare progress because health systems cannot test safe use interventions without continuously measuring the problem.^29^ Although a few studies have developed and tested outpatient trigger tools,^30–32^ none have been tested in children. We are currently leading stakeholders-driven research to address this gap.

Although this study provided a deep dive into the outpatient care of T1D, largely at home with the clinic’s support, this is a relatively small single-site study. Findings should be verified across multiple sites and in a larger population. As there is no similar work in children with T1D, findings in this study are similar to other single and multisite studies of medication errors among children and adults at home.

## CONCLUSIONS

Using SEIPs-based process mapping informed by multimodal methods to identify medication errors and treatment delays in children with T1D, we found errors were common. A significant burden of care exists at home for families of children with T1D; additional support from the healthcare system is needed to prevent errors. Better support for managing acute illness-associated hypo or hyperglycemia at home and more seamless communication between families at home and the diabetes clinic are areas rich in potentially high-yield interventions. Such solutions, co-produced with patients, families, and clinicians, can potentially improve the safety and quality of outpatient pediatric T1D developed by families at home.

## DISCLOSURE

Kathleen Walsh within the past 36 months has served as a consultant for Sanofi and Research Triangle Institute. All the other authors have no financial interests to declare in relation to the content of this article.
